# Spinal Anesthesia at the L2-L3 Versus L4-L5 Lumbar Vertebrae With Bupivacaine 0.5% (12.5 mg and 10 mg) Comparing Analgesia and Hemodynamic Stability: A Quasi-experimental Study

**DOI:** 10.7759/cureus.102927

**Published:** 2026-02-03

**Authors:** Aaisha Hanif, Syed Asadullah Jafri, Maryyam Hanif, Amjad Nadeem

**Affiliations:** 1 Anesthesiology, Combined Military Hospital, Karachi, PAK; 2 Anesthesiology, Combined Military Hospital, Lahore, PAK; 3 Anesthesia and Critical Care, Aga Khan University and Hospital, Karachi, PAK; 4 Anesthesiology, Khan Research Laboratories, Rawalpindi, PAK

**Keywords:** analgesia, bupivacaine, hemodynamic, pain, spinal

## Abstract

Objective: The objective of this study is to compare the outcomes of spinal block quality, hemodynamic stability, and adverse effects profile when giving spinal anesthesia using 10 mg and 12.5 mg of hyperbaric spinal bupivacaine at L2-L3 versus L4-L5 lumbar intervertebral space in bilateral inguinal hernia surgeries.

Materials and methods: It is a quasi-experimental study conducted at the anesthesia department of Combined Military Hospital (Malir Cantt), Karachi, from October 2022 to April 2023. A total of 220 patients were divided into the L2-L3 interspace group (n = 110) and the L4-L5 interspace group (n = 110) to be given spinal anesthesia with 0.5% bupivacaine 12.5 mg for the first unilateral hernia correction and to receive 10 mg of 0.5% bupivacaine for the second unilateral hernia correction eight weeks after the first procedure. Primary variables measured were time to complete the sensory block, total duration of the block, and time to first rescue analgesia after block regression. Secondary variables observed were perioperative hemodynamic parameters, including frequency of bradycardia and hypotension.

Results: The study concluded that using 12.5 mg of 0.5% bupivacaine in the L2-L3 space was associated with a higher frequency of bradycardia and hypotension while also providing the earliest onset and longest block regression time when compared with the L4-L5 space.

Conclusion: We conclude that the L4-L5 interspace is associated with adequate levels of spinal block quality and postoperative analgesia with less frequency of bradycardia and hypotension than the L2-L3 interspace and should be preferred in patients undergoing inguinal hernia surgery under spinal anesthesia.

## Introduction

Spinal anesthesia remains a desirable modality of anesthesia for lower limb surgeries. It is associated with fewer complications than general anesthesia, especially in the geriatric age group, but still provides effective pain relief and patient satisfaction [1]. It is reported in the literature that more than 80% of all lower limb surgeries are done under neuraxial anesthesia [2]. Despite its potential benefits, it is not without its adverse effects profile. The major side effects of spinal anesthesia are reported to be hypotension followed by bradycardia [3]. Other side effects include shivering, urinary retention, and nausea/vomiting [4]. These side effects are associated with the given dose, spinal level, and patient position. The onset of the block, along with sensory and motor blockade, is also dependent on the position and dose given [5]. The spinal block is given in the subarachnoid space below the conus [6]. The spinal space lies between two lumbar interspaces, and L2-L3, L3-L4, and L4-L5 are usually preferred for the neuraxial block. A gap in the literature shows that there is no consensus among anesthesiologists regarding which space is preferable as a standard guideline [7]. There is also disparity and diversity in the appropriate dose to be given in different spaces since both the spinal block space and block dose affect block outcomes.

One of the most commonly used drugs is bupivacaine. It belongs to the amide class of local anesthetic agents, and the spinal formulation is hyperbaric after addition of glucose to ensure a more even and dense sensory block [8]. The 0.5% formulation is commonly used in our setups. The rationale of our study is to review parameters of time to block onset, block recovery, and hemodynamic profile intraoperatively and postoperatively in patients undergoing lower limb surgeries by comparing L2-L3 and L4-L5 block interspaces using 12.5 mg and 10 mg of hyperbaric 0.5% bupivacaine.

## Materials and methods

This quasi-experimental study was carried out at the Department of Anesthesiology, Combined Military Hospital (Malir Cantt), Karachi, from October 2022 to April 2023 after approval from the ethical review board with letter no. 167/2022/Trg/ERC. Minimum sample size was calculated keeping the confidence interval at 95%, power of test at 80%, with the proportion of patients experiencing adverse effects of hypotension and bradycardia between the L2-L3 and L4-L5 interspace at 85% versus 68%, respectively [9]. Minimum sample size for one group came out to be 94 patients. We added a total of 110 patients in each group, making the total study sample of 220 patients. The method of sampling used was simple random sampling by lottery method. 

Inclusion Criteria

The inclusion criteria are as follows: the American Society of Anesthesiologists (ASA) I and II patients, aged 18-65 years, of both genders with bilateral inguinal hernia planned for unilateral correction of inguinal hernia, first the right and then the left side, which are eight weeks apart.

Exclusion Criteria

The exclusion criteria are as follows: patients unwilling to undergo spinal anesthesia, refusal to be included in the study, patients with low ejection fraction (<40%), patients with severe respiratory compromise, patients with known allergy to bupivacaine, patients with deranged coagulation profile as a contraindication to spinal, and those patients with patchy or failed spinal anesthesia and conversion to general anesthesia.

The study included all patients fulfilling the inclusion criteria. The patients were divided into two groups. Group A included the patients receiving spinal anesthesia in the L2-L3 interspace (n = 110), and group B included the patients receiving spinal anesthesia in the L4-L5 interspace (n = 110). These groups were to receive spinal anesthesia with 0.5% hyperbaric bupivacaine 12.5 mg for the first unilateral hernia correction and to receive 10 mg of 0.5% hyperbaric bupivacaine for the second unilateral hernia correction eight weeks after the first procedure. The method of sampling was simple random sampling via the lottery method. Informed written consent was taken from all patients, and they were counselled to follow up for correction of the second procedure as a part of the study protocol. Patients lost to follow-up were excluded from the study protocol. 

Primary variables measured were time to complete the sensory block, total duration of the block, and time to first rescue analgesia after block regression. Secondary variables observed were perioperative hemodynamic parameters, including frequency of bradycardia and hypotension.

On the day of the procedure, patients from both groups received 500 ml of normal saline 15 minutes before being shifted to the operating room. Standard monitoring as per ASA protocols, including noninvasive blood pressure, heart rate, pulse oximetry and ECG, was attached to the participants in both groups. All patients received an antiemetic protocol in the form of IV dexamethasone 4 mg and IV ondansetron 8 mg stat, 10 minutes before the procedure. Ondansetron was also given to suppress the Bezold-Jarisch reflex for better hemodynamic stability. Antibiotic prophylaxis was given to all patients with IV ceftriaxone. Spinal anesthesia for the first unilateral inguinal hernia repair procedure was given with a dose of 0.5% bupivacaine 12.5 mg, and a dose of 10 mg for the second hernia correction done eight weeks after the first procedure in all patients. Group A received the dose in the L2-L3 interspace, while group B received the dose in the L4-L5 interspace. Spinal anesthesia was administered by a consultant anesthesiologist under strict aseptic measures in the sitting position in all patients using a 27 G pencil point needle [10]. Sensory blockade till the T10 dermatome level was confirmed by loss of sensation to cold ethyl chloride spray and pin prick in the midline and bilaterally below the umbilicus. Once a successful block was achieved, the surgery was then continued. Hemodynamic parameters, including heart rate, mean arterial pressure, and saturation levels, were checked immediately after the spinal administration and then after every 15-minute interval till the end of surgery by a resident anesthesiologist in the operating room, unaware of the study protocol or its outcomes. Hypotension was defined as a mean arterial blood pressure of less than 60 mm Hg, and bradycardia was defined as a heart rate of less than 50 beats per minute [11,12]. Hypotension was treated with a bolus of 100 mcg of IV phenylephrine. Bradycardia at any point was treated with IV atropine 0.5 mg bolus. For arterial oxygen saturation of less than 92%, supplemental oxygen was provided with nasal prongs. At the end of the hernia correction and mesh repair, patients were shifted to the recovery room and subsequently to the ward. Block regression was checked hourly till sensations returned completely in all patients assessed by a resident anesthesiologist on duty in the recovery and subsequently in the ward. Time for first rescue analgesia was assessed using the visual analog scale (VAS) by the resident on duty and was given when pain scores were above 6 on the VAS [13].

Demographic data, including age, weight, and gender, were statistically described in terms of mean ± SD, frequencies, and percentages when appropriate. Continuous variables of age and weight were checked for normality and were normally distributed. Mean values for mean block onset, regression time, and time to first dose of rescue analgesia were expressed as mean ± SD and compared using the independent samples t-test. Adverse effects of hypotension and bradycardia were expressed as frequency and percentage and compared using the Chi-square or Fisher's exact test as appropriate. A p-value of <0.05 was considered statistically significant. All statistical calculations were performed using IBM SPSS Statistics for Windows, Version 26 (Released 2018; IBM Corp., Armonk, New York, United States).

## Results

**Figure 1:**
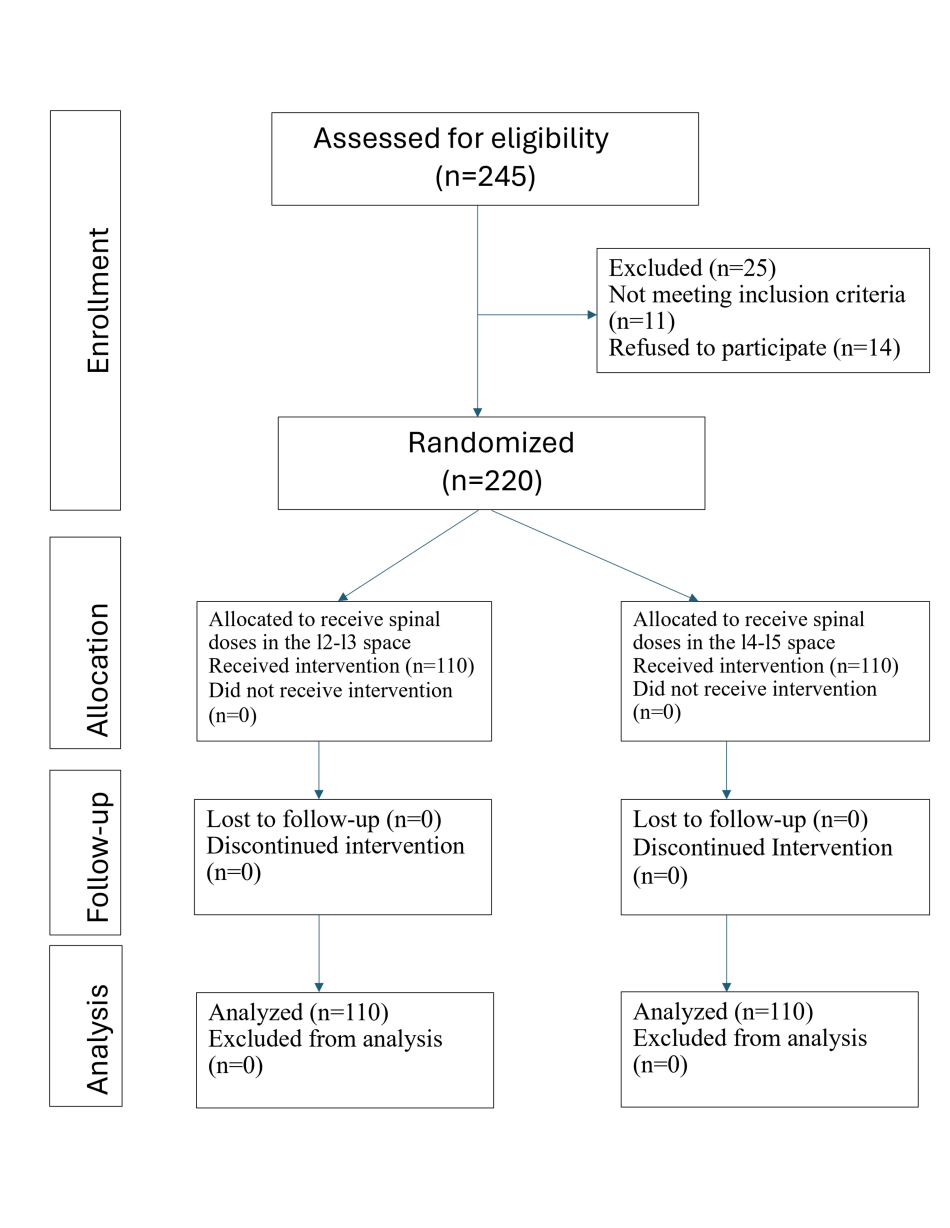
Consort diagram

A total of 220 patients were analyzed in the final study protocol, divided into group A (n = 110) receiving spinal doses in the L2-L3 space and group B receiving spinal doses in the L4-L5 space (Figure 1). Table 1 shows a comparison of the demographic and clinical variables in both groups.

**Table 1:** Comparative analysis of demographics and clinical variables

	Group A	Group B	Test statistic	p-value
	(n = 110)	(n = 110)		
Age (years)	47.79 ± 6.41	46.57 ± 6.67	1.38	0.169
Weight (Kg)	73.80 ± 3.75	74.34 ± 3.52	−1.10	0.276
Gender				
Male	110 (100%)	110 (100%)		
Female	0 (0%)	0 (0%)		
Duration of surgery (minutes)	78.82 ± 6.45	77.79 ± 5.97	1.23	0.222

Spinal block parameters were assessed in the first and second surgery and a comparison of the results in both groups is shown in Table 2. For both first and second surgery, the mean time for effective block and the mean time for block regression show statistically significant differences in both groups (p-value < 0.005), and the difference of mean time to first dose rescue analgesia was not statistically significant (p-value = 0.764 for first surgery and p-value = 0.563 for second surgery) (Table 2).

**Table 2:** Comparative analysis of block onset, block regression and rescue analgesia between group A and group B

Variable	Group A	Group B	Test statistic	p-value
	(n = 110)	(n = 110)		
Spinal block with 12.5 mg Of 0.5% bupivacaine				
Time for effective sensory block (T8) (minutes)	5.16 ± 0.67	6.72 ± 0.62	-17.92	<0.001
Time for sensory block regression (S1) (minutes)	232.89 ± 7.93	202.98 ± 9.03	26.1	<0.001
Time to first dose rescue analgesia (minutes)	336.87 ± 17.44	336.15 ± 18.02	0.3	0.764
Spinal block with 10 mg of 0.5% bupivacaine				
Time for effective sensory block (T8) (minutes)	7.03 ± 0.73	8.04 ± 0.74	-10.19	<0.001
Time for sensory block regression (S1) (minutes)	202.93 ± 8.79	190.80 ± 7.95	10.73	<0.001
Time to first dose rescue analgesia (minutes)	336.79 ± 17.40	335.38 ± 18.65	0.58	0.563

Hemodynamic parameters during the first surgical correction with 12.5 mg of 0.5% bupivacaine showed increased frequency of bradycardia even after 45 minutes following the institution of spinal anesthesia in group A (p-value < 0.005), and the difference was statistically significant even after 45 minutes (p-value = 0.014 for after 45 minutes). The incidence of hypotension was also higher in group A before 45 minutes (p-value < 0.001), and even after 45 minutes, the difference was statistically significant (p-value = 0.017). 

For the second surgery with the utilization of decreased dosage (10 mg of 0.5% bupivacaine), there was a difference in the frequency of bradycardia between the two groups (p-value = 0.017 immediately after spinal and p-value = 0.002 after 15 minutes), and after 30 minutes, there was no statistically significant difference between the findings for bradycardia in both groups. There was no statistically significant difference among the findings for hemodynamic variables in both groups (Table 3).

**Table 3:** Comparative analysis of hemodynamic profile between group A and group B

Variable	Group A (n = 110)	Group B (n = 110)	Test statistic	p-value
Spinal block with 12.5 mg of 0.5% bupivacaine				
Frequency of bradycardia				
Immediately after spinal	82 (74.5%)	45 (40.9%)	24.14	<0.001
After 15 minutes	68 (61.8%)	40 (36.4%)	13.26	<0.001
After 30 minutes	40 (36.4%)	21 (19.1%)	7.35	0.004
After 45 minutes	23 (20.9%)	10 (9.1%)	5.13	0.014
Frequency of hypotension				
Immediately after spinal	87 (79.1%)	46 (41.8%)	30.42	<0.001
After 15 minutes	70 (63.6%)	37 (33.6%)	18.63	<0.001
After 30 minutes	43 (39.1%)	19 (17.3%)	11.88	<0.001
After 45 minutes	25 (22.7%)	11 (10.0%)	5.61	0.017
Spinal block with 10 mg of 0.5% bupivacaine				
Frequency of bradycardia				
Immediately after spinal	79 (71.8%)	62 (56.4%)	5.06	0.017
After 15 minutes	60 (54.5%)	30 (27.3%)	15.81	0.002
After 30 minutes	30 (27.3%)	24 (21.8%)	0.61	0.347
After 45 minutes	12 (10.9%)	12 (10.9%)		1
Frequency of hypotension				
Immediately after spinal	45 (40.9%)	39 (35.5%)	0.48	0.405
After 15 minutes	25 (22.7%)	30 (27.3%)	0.39	0.436
After 30 minutes	15 (13.6%)	11 (10.0%)	0.39	0.404
After 45 minutes	05 (4.5%)	11 (10.0%)	1.69	0.119

## Discussion

The study concluded that using 12.5 mg of 0.5% bupivacaine in the L2-L3 space was associated with the highest frequency of bradycardia and hypotension, while also providing the earliest onset and longest block regression time when compared with the L4-L5 space. When comparing 10 mg of 0.5% bupivacaine, bradycardia and hypotension were less than the 12.5 mg dose, with comparative frequencies of hypotension and bradycardia between the L2-L3 and L4-L5 interspaces when measured pre- and postoperatively. While the frequency of adverse effects was less, the onset of block was longer, and block regression time was shortened compared to the 12.5 mg dose. It is pertinent to note that the time to the first dose of rescue analgesia was the same between both interspaces. 

Critical analysis with local and international literature shows that in a study carried out by Zhang et al., patients undergoing cesarian section surgeries had a faster onset of action when spinal doses were given in the L2-L3 interspace, but with an associated higher incidence of bradycardia and hypotension at 79% and 72%, respectively. This is in line with findings of our study where the frequency of adverse effects in the L2-L3 interspace were 74.5% and 79%, respectively [14]. In another study carried out by Kietzmann et al. in a remote hospital using spinal anesthesia for lower abdominal surgeries, the L4-L5 was preferred due to less frequency of hypotension and bradycardia. This was associated with a longer onset of action when using higher spaces, but the clinical difference was negligible (five minutes to 7-8 minutes) [15]. These results were in accordance with the findings of our study, where the difference between block onset and spinal doses given at the two interspaces ranges between 1.5 and two minutes, which becomes negligible in the clinical setting for elective procedures. In another study by Manouchehrian et al., studying the hemodynamic parameters when giving spinal anesthesia in the L2-L3 versus L4-L5 lumbar space concluded that block level at the L4-L5 space provided adequate spinal block quality with good postoperative recovery times and less frequency of bradycardia and hypotension and was recommended to be preferred in cesarian section patients where the chances of hypotension and bradycardia are higher due to deranged physiology and compression of the major veins due to the gravid uterus [16].

In a study done by Asghar et al. in Islamabad, the authors concluded that lower spaces are associated with adequate analgesia and less hemodynamic compromise and should be preferred, especially in compromised patients. Higher spaces should only be used in case there is difficulty in the spinal cord in the lower levels [17]. These conclusions and recommendations are also proposed by our study findings, where the levels of postoperative analgesia requirement is same between both spaces, but since lower spaces offer better hemodynamic stability, they should be considered as the first-line spinal approach in patients. In another local study for pediatric patients undergoing spinal anesthesia, lower spaces offer better analgesia, fewer chances of spinal failure, and better postoperative recovery and are preferred over higher spaces [18].

The limitations of our study are that the study is only done for inguinal hernia repair. Secondly, we carried out this study using two different doses in the two interspaces for ASA I and ASA II patients only. More studies on patients undergoing different surgical procedures with established hemodynamic compromise need to be done to form guidelines for the best possible approach.

## Conclusions

We conclude that the L4-L5 interspace is associated with adequate levels of spinal block quality and postoperative analgesia with less frequency of bradycardia and hypotension than the L2-L3 interspace and should be preferred in patients undergoing inguinal hernia surgery under spinal anesthesia.
